# German Parents and Educators of Two to Four-Year-Old Children as Informants for the Strengths and Difficulties Questionnaire (SDQ)

**DOI:** 10.1007/s10578-024-01767-2

**Published:** 2024-10-03

**Authors:** Simone Dubiel, Franziska Cohen, Yvonne Anders

**Affiliations:** 1 Humboldt University Berlin, Berlin, Germany; 2 Department of Childhood Education, Institute of Education Science, University of Education Freiburg, Freiburg, Germany; 3 Department of Early Childhood Education, University of Bamberg, Bamberg, Germany

**Keywords:** Rater agreement, Strengths and difficulties questionnaire, Operations triad model, Multitrait-multimethod matrix, Social-emotional behavior

## Abstract

Screeners are used in early intervention and early childhood education and care programs to identify children’s potential need for further evaluation and diagnostics. The Strengths and Difficulties Questionnaire (SDQ) is a brief behavioral screening instrument that can be completed by both parents and educators to assess the social and emotional traits of children. However, multiple informants’ reports vary. In this study, the extent to which parents’ (*n* = 241) and educators’ (*n* = 157) differ and agree in their assessments of children aged 3.5 years on average, was examined. *T*-tests were used to examine differences between informants and correlations within a multitrait-multimethod matrix (MTMM) in their agreement. Results showed moderate to high levels of rater agreement ranging from *r* = .35 and *r* = .53 on the five subscales of the SDQ. We found that hyperactivity, peer relationship problems, and prosocial behavior vary due to meaningful reasons, e.g., the home vs. pre-school setting, and the informant’s relationship towards the child. Hyperactivity seems to be relatively consistent across settings. Methodological variations might explain differences in emotional symptoms and conduct problems. Considering ratings from multiple informants outlines a more comprehensive view of children’s behavior and should be preferred over single-informant research designs.

Understanding young children’s social-emotional behavior unveils a crucial factor in shaping subsequent academic, social, and educational outcomes [1]. Screeners, pivotal in tailoring interventions and evaluating potential risks affecting infants’ development, are widely used tools across various programs, interventions, and clinical diagnostics are used to collect data, support interventions and identify problems in young children’s behavior. The *Strengths and Difficulties Questionnaire (SDQ)* is one of the most widely used screening instruments for those purposes.

To obtain a robust and holistic picture of a child, screeners, and comprehensive assessments and diagnoses should be collected from more than one informant [2]. In many Early Childhood Education and Care (ECEC) programs and clinical diagnostics, parents and teachers are those informants [3]. Parents have unique insight into a wide range of their child´s behavior, especially before entering a childcare facility [4, 5]. Teachers, on the other hand, observe children usually without child-parent influences and in the surroundings of peers [5]. Both sets of informants contribute essential information about children’s social and emotional behaviors [6–8].

Consequently, it seems necessary to include multiple informants when evaluating children – especially since parents and educators^1^ do not necessarily agree [3, 11]. On the contrary, discrepancies between informants are one of the most robust findings in clinical child research [9]. The meta-analysis by Achenbach et al. [2] established that the agreement of different types of informants only had a moderate mean correlation of *r* = .28. De Los Reyes et al. [10]conducted a follow-up meta-analysis of 341 cross-informant studies to assess children’s mental health and came to similar conclusions [10]. Therefore, we need a better understanding of how parents and educators perceive and report young children´s social and emotional behavior to facilitate interpretations across different perspectives [7, 8, 11].

## Predictors for Rater Agreement

Achenbach et al. [2]revived the debate on whether situational factors or personality characteristics mainly determine behavior. They asked whether and how much consistency can be found between informants and whether it varies with the type of informant or the child’s characteristics, such as age, sex, type of problems, or degree of deviance. We will summarize some research findings for determinants of low rater agreement in the following:

(1) Children behave differently in different settings, mitigating criticisms of a lack of validity and reliability in research practice [2, 12]. Achenbach et al. [2] concluded this after examining 269 samples from 119 studies in a meta-analysis. The samples included children and adolescents from 1.5 to 19 years of age. The study focused on surveys of behavioral and emotional problems in children. The weighted mean Pearson r between informants playing similar roles towards the child was 0.60, whereas the weighted mean *r* between ratings by different informants dropped to 0.28 [2]. They concluded that both the informant’s role regarding the child and the settings where children spend their time play essential roles in low-rater agreement. They stated that certain behaviors or symptoms could occur or be absent in different constellations of environment and rater-child interaction. Thus, assessing the full range of a child’s behaviors by a single rater is difficult [2, 13]. Cai et al. [14] also recommend considering the informant’s role and the specificity of parents’ and educators’ environments when interpreting findings. They examined parents’ and educators’ agreement on individual problem behaviors with a sample of 3-year-old preschool children (*N* = 614) from low-income and predominately African-American families. Educators (as consistent with their roles) most often indicated problem behaviors closely related to children’s learning (can’t sit still), classroom management (quickly shifts), and peer relationships (hits others). Parents in the home setting most frequently reported behaviors maintaining the household (want things now) and raising emotionally healthy children (feelings easily hurt).

(2) Rater agreement seems to be higher when assessing externalizing behavior problems in contrast to internalizing behavior problems of children [14, 15]. Externalizing behavior problems (e.g., aggression and rule-breaking behaviors) are easier to observe for informants than internalizing behavior problems (e.g., anxiety or depression) [2, 10, 16, 17]. In consequence, rater agreement is higher for observable than for unobservable symptoms [9]. However, parents perceive internalizing problems as typically more covert symptoms, better than educators [10]. Further Martinsone et al. [18] found, that parents rated higher problem behavior for their children than educators while Murray et al. [19] and Bergold et al. [15] found that parents rated in all domains higher than educators.

(3) Rater agreement regarding the age of the children corresponds more for younger children than for adolescents [5, 20]. De Los Reyes and Kazdin [9]concluded that informants generally spend more time with children of younger age, resulting in higher agreement consistency. Another explanation is that young children’s behavior is more constrained across settings with comparable situations [9]. Older children tend to be assessed with more prosocial behavior than younger children [21].

(4) Some studies found relations between the child’s gender and informant perception in clinical communities and school-based populations [21–23]. Other studies found no gender effect [5, 24]. Although there have been findings in both directions, the overall view suggests that the child´s gender is not related to informant agreement. However, a gender effect may occur in special populations [9].

(5) In addition, educators as informants are influenced by their expectancies, subjective perceptions, knowledge, and opportunities for comparison in their judgment [25]. Educators evaluate children’s educational needs according to their achievement levels. As a result, a child may be considered in need of education in one context but not in another, depending on the local judgments. Therefore, educators’ and teachers’ opportunities for comparison are related to children’s needs based on the prevailing criteria in the respective environment [26]. A study by Chwastek et al. [21] on a German sample of refugee children demonstrated that additionally, the beliefs and attitudes of professionals, as well as their self-efficacy and their professional experiences, can influence assessments of children’s social-emotional behavior.

(6) Parent-child relationships and parent characteristics play a role when assessing children [9, 27–30]. For example, Vaughn et al. [31] examined the discriminant validity of the Carey Infant Temperament Questionnaire with 189 first-time mothers who completed a battery of psychological tests before and after giving birth. They distinguished babies being temperamentally ‘difficult’ from those diagnosed as ‘easy’ based on their mother’s statements three and six months after the baby was born. They concluded that there might equally be a ‘difficult child syndrome’ or a ‘difficult mother syndrome’. Mothers of ‘difficult’ infants were less likely to present a socially desirable picture of themselves [31]. The study by Van der Oord et al. [32] discovered that parenting stress affected parental consensus on attention deficit hyperactivity disorder and oppositional defiant disorder. Further, it is considered, that parental reflective functioning, the parent’s ability to understand a child’s behavior in social settings, protects children from social-emotional difficulties and shapes their long-term development [33]. Parental reflective functioning is linked to their children´s mental health and development and adults´ mental health [27]. It was found that poor maternal prenatal reflective functioning is related to high infant physical aggression [34]. In contrast, mothers did not report increased physical aggression when they had both low prenatal RF-skills and were less sensitive during the interaction with their infant [34].

Regarding the parent-child relationship Stone et al. [35] showed that higher SDQ levels in younger age children were associated with more inadequate parenting behavior and stress of the parents when children became older. Michels et al.‘s [36] study highlighted that authoritative parenting correlated with more parent-educator agreement, while dysfunctional parenting behaviors like over-reactivity and low-caring attitudes were linked to more informant discrepancies due to potential differences in recognizing and addressing child behaviors and emotions.

To sum up, the informants’ role and setting are predictive for ratings as well as the kind of assessed behavior (externalizing vs. internalizing). Educators and teachers are influenced by their expectancies, subjective perceptions, knowledge, and opportunities for comparison in their judgment. Parents´ statements are influenced by the relationship with the child and the parents´ characteristics. Results regarding children’s age and gender seem to be inconsistent. The advantages and challenges of using parents and educators as informants must be well-balanced to obtain meaningful interpretations of findings. Because there is no standard for measuring the true extent of a child’s potential disruption, informant agreement cannot be used to conclude the validity of a particular informant’s assessment [9].

## The Operations Triad Model (OTM)

The challenge lies in addressing the discrepancy in informant reports, treating them as both a confounding factor requiring methodological solutions [37, 38] and valuable information [3]. These considerations lead to diverse data processing approaches. A contradiction arises when researchers view discrepancies as valuable while using methods treating them as confounding factors [39]. Despite this awareness, there is a lack of standardized procedures, highlighting a research gap. De Los Reyes et al. [39] introduced the Operations Triad Model (OTM), a decision tree for handling different data outcomes, particularly diverging ones. It is a guideline for interpreting outcome results for converging findings, diverging findings, or an undefined mixture of diverging findings and methodological reasons, in which divergent ratings are classified into meaningful and non-meaningful divergences. Based on previous findings, we assume for our study the rater agreement between parents and educators to be moderate due to meaningful reasons and therefore explain in more detail diverging and compensating operations.

### Diverging and Compensating Operations

Converging operations are of little help when divergent research findings are suspected [39]. As mentioned earlier, divergent findings can occur for various reasons. In classical test theory, divergence reflects measurement error – which can be one reason for inconsistency; however, meaningful differences can be another. Therefore, divergences are divided into diverging and compensating operations within the Operations Triad Model [40].

The concept of diverging operations is applicable under the following conditions: The observed behavior of the child varies (e.g., at home vs. preschool), and the discrepancy between parents’ and educators’ statements reflects this in a meaningful way because of the behavior of the child. Compensating operations, in contrast to divergent operations, indicate that discrepancies in findings are not due to meaningful reasons. In this case, reliability and validity are of great relevance to distinguish between method effects and reasonable divergence [39]. Discrepancies may be caused by methodological differences and/or reliability or validity issues. In the case of compensating operations, reconsidering statistical methods is advisable if the discrepancy is assumed to result from a methodological difference in otherwise reliable and valid findings. If findings are neither valid nor reliable, one group of informants may be excluded from further processes [39].

Before collecting data, hypotheses should be made regarding whether findings will converge or diverge. When assuming convergent findings, categories of ‘and’ and ‘or’ can be applied [39]. The ‘and’ classification would imply that parents and educators assess the child in the same way. For the condition ‘or’, the two informants would be considered separately [41]. As this method has shown high predictive power, Goodman et al. [16] approve of this procedure, especially in clinical contexts. Suppose predictive power is not the central aim of a survey, and the assumption of divergent findings based on prior empirical studies is made, considerations about reliability and validity should apply [39].

## Research Question

The reason and the extent of low raters’ agreement are unknown factors and therefore, can lead to uncertainty in planning interventions and taking decisions for clinicians, educators and researchers [6, 39]. However, variability can equally be used as a possible source of meaningful information [42, 43].

To date, we are not aware of any study that examines parents’ and educators’ statements of young children’s behavioral strengths and difficulties based on the theoretical considerations of the OTM. Previous studies on this topic focus on low rater agreement using mean value comparisons, emphasizing the validity of the SDQ [11], or challenges to addressing discrepant perceptions. In this study, we combine these approaches and aim at the following two research questions:

1. To what extent do parents and educators differ in their ratings of the SDQ?

Hypothesis 1.1: The observed characteristics of parents and educators differ on average.

2) What is the relative proportion of traits and method-specific influences on parents’ and educators’ agreement on ratings of the SDQ?

Hypothesis 2.1: The individual observed traits are correlated.

Hypothesis 2.2: The observed traits can be distinguished across the perspectives of parents and educators.

Hypothesis 2.3: The survey instrument is equally suitable for both parents and educators.

Based on previous findings, we assume the rater agreement between parents and educators to be moderate due to meaningful reasons. Therefore, in a subsequent step, we apply the quality criteria of diverging and compensating operations of the Operations Triad Model.

## Method

### Design

We examined data from the cross-sectional study AQuaFam, based in North Rhine-Westphalia, Germany. The data were collected using questionnaires. The surveys of parents took place at the families’ homes by conducting standardized interviews and those of educators at the children’s childcare settings. Data represents an unremarkable, non-clinical sample.

### Sample

The subset of parents comprised a sample size of *N* = 241 with children having an average age of 41months (min = 23 months, max = 53 months). 54.1% of the children were male, 21% had no siblings. Further 91.7% of mothers and 5.4% of fathers filled out the questionnaire about their children. In addition, another 2.9% filled out the questionnaire together (mothers and fathers after joint consultation). Since our questionnaire was aimed at assessments of parents without distinguishing between mothers and fathers, all parental statements were included in the analysis. The mothers were, on average, 34.37 (min = 21, max = 47) years old. 62.4% graduated from the highest track of German secondary education, and 45.1% of the mothers achieved an academic degree. 69.5% of the mothers were employed, while 12.9% were non-employed. For 27.8% of the mothers, German was not their native language. The parent sample and thus also the child sample is predominantly European German. We have no further detailed information on which languages are spoken in the family. The migrant population in the region is mainly Turkish, Polish and Arabic-speaking [44].

In addition, the behavior of the children was assessed by 157 educators in their pre-school facilities. Approximately half of the educators confirmed they knew the child well; 28.1% even felt they knew the child well. 98.1% of the educators had known the child for a year or more. 61.4% of the children attended a daycare center (*Kindertagesstätte*), and 1.2% of the children attended family day care (*Kindertagespflege*) provided by a childminder (*Tagesmutter*).

The time span between the parent and educator assessments was within one month for 78% of the children (M = 1.21, SD = 1.94, min = 0, max = 8).

The children for whom an educator assessment is available differ in a few characteristics compared to those without educator assessments. The children are older, the families’ income is higher, and a family language other than German is less frequently spoken. The mother’s educational background and the home learning environment are not significantly different. Further, the reported assessments of the SDQ subscales are the same for both groups of parents. Only the hyperactivity scale is lower in the group with educator assessments.

### Measures

We used the SDQ [45] to assess the children’s social and emotional behavior. The SDQ is a brief behavioral screening questionnaire for children and adolescents between the ages of approximately 3 and 16, widely used by researchers, clinics, and educational psychologists. It includes five subscales with five items each on the psychological dimensions: *emotional symptoms*,* conduct problems*,* hyperactivity*,* peer relationship problems*,* and prosocial behavior*. Parents and educators reported on the questionnaire of the SDQ once. Items were rated with ‘not true’ = 0, ‘somewhat true’ = 1, and ‘certainly true’ = 2. It takes about five to ten minutes to complete the questionnaire. For the survey, we used the German version of the SDQ. Klasen et al. stated that the factor structure of the German SDQ is similar to the original English-language version and can be used as a screening tool in normal populations (e.g., schools and homes).

Goodman et al. [16] analyzed the convergent and discriminant validity of the SDQ for parents, school teachers, and adolescents, based on a representative sample of 5–16 year-olds in England. As a result, they propose summarizing emotional symptoms and peer relationship problems to internalizing behaviors’ characteristics. Conduct problems, hyperactivity and prosocial behavior are externalizing behaviors’ characteristics. They concluded that the broader internalizing and externalizing SDQ subscales are beneficial for analyzing low-risk children. To examine the relations between behavioral traits, it seems reasonable to retain all five subscales [16]. The items were reversed and aggregated to subscales, as advised by Goodman [45]. Each subscale could range between 0 and 10.

### Analysis

To answer the first research question, to what extent parents’ and educators’ ratings differ, a two-tailed paired Student’s *t*-test was conducted with the aggregated subscales of the SDQ to determine the mean differences of the parents and the educators.

To determine the relative proportion of traits and method-specific influences on parents and educators’ agreement on the SDQ, the multitrait-multimethod matrix (MTMM) as a method of validation for psychological measures, developed by Campbell and Fiske [46], was applied. The matrix provides a variety of information, particularly the reliability and validity diagonal. Reliability coefficients via Cronbach’s alpha for each subscale and rater were calculated. We conducted a latent structural equation analysis for each group (parents and educators) according to the theoretically given structure of Goodman. We calculated the latent constructs’ estimated correlations using the TECH4 command in Mplus 7.4 [47]. We accounted for the ordered structure of the indicators using polychoric correlations [48]. We needed to exclude two items from the parental questionnaire causing a not positive definite residual covariance matrix: pbullied (peer relationship problems) and pworries (emotional symptoms). We interpreted the coefficients based on the following guidelines: *r* ≥ .10 for small effects, *r* ≥ .30 for medium effects, *r* ≥ .50 for large effects [49]. To account for missing data, Full Information Maximum Likelihood (FIML) estimation was employed for the MTMM analysis. For the two-tailed paired Student’s *t*-test, only paired data (parents and educators) were used so that there were no missing data.

Further, a multitrait-multimethod matrix consists of correlation triangles. In the present study, parents and educators represent different methods and the subscales/constructs of the SDQ have different traits. The correlation triangles provide information about the relationship between traits and methods. To achieve good construct validity, correlation values of the same traits, but different methods (we refer to this combination as monotrait-heteromethod correlations) should be significantly different from zero and sufficiently high. Correlations coefficients for different traits, but the same methods (heterotrait-monomethod correlations) are supposed to be on a low level. Campbell and Fiske [46] suggested four considerations for multitrait-multimethod matrices. The first consideration focuses on convergent validity. The following three considerations concern the test instruments’ discriminant validity.

1. The validity diagonal (values between the dashed triangles in Table 2) contains correlations of the same characteristics (subscales of the SDQ) but different methods (parents and educators). Coefficients on the validity diagonal should be significantly different from zero and sufficiently high.
2. Values on the validity diagonal should be higher than those of the heterotrait-heteromethod triangles (values within dashed triangles). The consideration arises from the idea that values should be higher for monomethod-heterotrait correlations than those representing different traits and different methods.
3. Values for monotrait-heteromethod correlations should be higher than heterotrait-monomethod correlations. Therefore, values on the validity diagonal are compared with values in the heterotrait-monomethod triangles (values in solid-lined triangles).
4. Campbell and Fiske’s [46] fourth consideration implies that the triangles’ values should be at approximately the same level. The strength of the correlation is represented by different shades of gray.

Additionally, we calculated the fit indices for each model (parents and educators). Consequently, two separate structural equation models (parents and educators), with the five subscales/constructs of the SDQ as latent factors, were analyzed. We report the root mean square error of approximation (*RMSEA*), the comparative fit index (*CFI*), and the Tucker-Lewis Index (*TLI*) in the results section. *RMSEA*s that are no greater than 0.08, and *CFI*s and *TLI*s of 0.90 or higher, suggest an acceptable model fit [50].

## Results

### Differences Between Parents’ and Educators’ Ratings

Table 1 shows the descriptive findings and the results for the paired *t*-test of the five SDQ subscales for parents compared to educators. Significant mean differences can be found for the subscale prosocial behavior, parents reporter higher prosocial behavior (*M* = 7.68, 1.66) for their children than educators (*M* = 6.86, *SD* = 2.38), *t*(154) = 4.11, *p* = .00). In contrast, we found no significant mean differences between parents and educators for the subscales: emotional symptoms, conduct problems, hyperactivity, and peer relationship problems.

**Table 1:** Descriptive statistics of the SDQ with Mean differences of parents’ and educators’ ratings

	Parents		Educators		t-test			
Scale	M	SD	M	SD	M	SD	T	*p*
Emotional Symptoms	1.46	1.42	1.26	1.55	0.10	1.78	0.68	0.50
Conduct Problems	2.39	1.58	2.01	2.15	0.25	2.36	1.30	0.19
Hyperactivity	3.28	1.97	3.33	2.59	− 0.30	2.55	− 0.1.45	0.15
Peer Relationship Problems	1.43	1.64	1.31	1.65	− 0.06	1.91	− 0.38	0.71
Prosocial Behavior	7.68	1.66	6.86	2.38	0.79	2.38	4.11	0.00**

*Note*: Parents: *n* = 241, educators: *n* = 157. Response categories: *not true* = 0, *somewhat true* = 1, *certainly true* = 2, the scales can reach a maximum of 10, as *certainly true* holds the value 2 and each subscale contains 5 items (5 times 2). *t*-test: *n* = 157 pairs; paired difference test for the five subscales of the SDQ. *p* = 2-sided significance level *p < .05. **p < .01

### Agreement of Parents’ and Educators’ Ratings

Table 2 presents the results of the multimethod-multimethod matrix of influences on parents´ and educators´ rating agreement. The reliability diagonal (values in brackets) shows Cronbach’s alpha values for all combinations of the same trait (subscales of the SDQ) and different methods (parents vs. educators). Cronbach’s alpha values for educators range from *α* = 0.39 (conduct problems) to *α* = 0.82 (prosocial behavior). Cronbach’s alpha values for parents range from *α* = 0.51 (conduct problems) to *α* = 0.70 (hyperactivity). In the following, we describe our findings according to the four considerations of Campbell and Fiske [46].

1. On the validity diagonal (values between the dashed triangles), correlation values range from *r* = .35 to *r* = .53. All values on the validity diagonal show moderate correlations, except for prosocial behavior (*r* = .53) and peer relationship problems (*r* = .51), which show high correlations.
2. Values on the validity diagonal should be higher than those of the heterotrait-heteromethod triangles (values within dashed triangles). Findings show relatively high correlations between conduct problems_educators_ and hyperactivity_parents_ (*r* = .39) and between conduct problems_educators_ and prosocial behavior_parents_ (*r* = − .39). The subscale prosocial behavior_educators_ correlates quite highly with conduct problems_parents_ (*r* = -38), hyperactivity_parents_ (*r* = − .41), and peer relationship problems_parents_ (*r* = − .35). As a result, the correlations given here are on par with the monotrait-heteromethod values on the validity diagonal: emotional symptoms (*r* = .42) and conduct problems (*r* = .35), which violates the second consideration.
3. Values on the validity diagonal are compared with values in the heterotrait-monomethod triangles (solid-lined triangles) because monotrait-heteromethod correlations should be higher than heterotrait-monomethod correlations. Within the heterotrait-monomethod triangle of parental responses, notable findings are correlations of emotional symptoms with peer relationship problems (*r* = .56), conduct problems with hyperactivity (*r* = .55), and peer relationship problems with prosocial behavior (*r* = − .55). Within the educators’ heterotrait-monomethod triangle, notable findings are correlations of emotional symptoms with peer relationship problems (*r* = .52), conduct problems with hyperactivity (*r* = .63), conduct problems with prosocial behavior (*r* = − .73), hyperactivity with prosocial behavior (*r* = − .63) and peer relationship problems with prosocial behavior (*r* = − .53). Some heterotrait-monomethod correlation coefficients are higher than the corresponding monotrait-heteromethod correlations, violating the third consideration.
4. Campbell and Fiske’s [46] fourth consideration imply that the triangles’ values should be approximately identical. The inner structure of the values only partially shows the same pattern. While the coefficients of the educators’ triangle show the highest correlations between prosocial behavior and conduct problems (*r* = − .73) as well as hyperactivity (*r* = − .63), the parents’ triangle of the same pairs of correlation coefficients reflects this only partly (*r* = − .52 and *r* = − .32). The heterotrait-heteromethod block values differ even more (*r* = − .38 and *r* = − .41) and (*r* = − .19 and *r* = − .39). In contrast to all other correlations, the value 0.19 is not significant. Table 3 gives an overview of the distribution of significant coefficients in the multitrait-multimethod matrix, showing the highest correlation coefficients lie within the heterotrait-monomethod triangles.

**Table 2:**
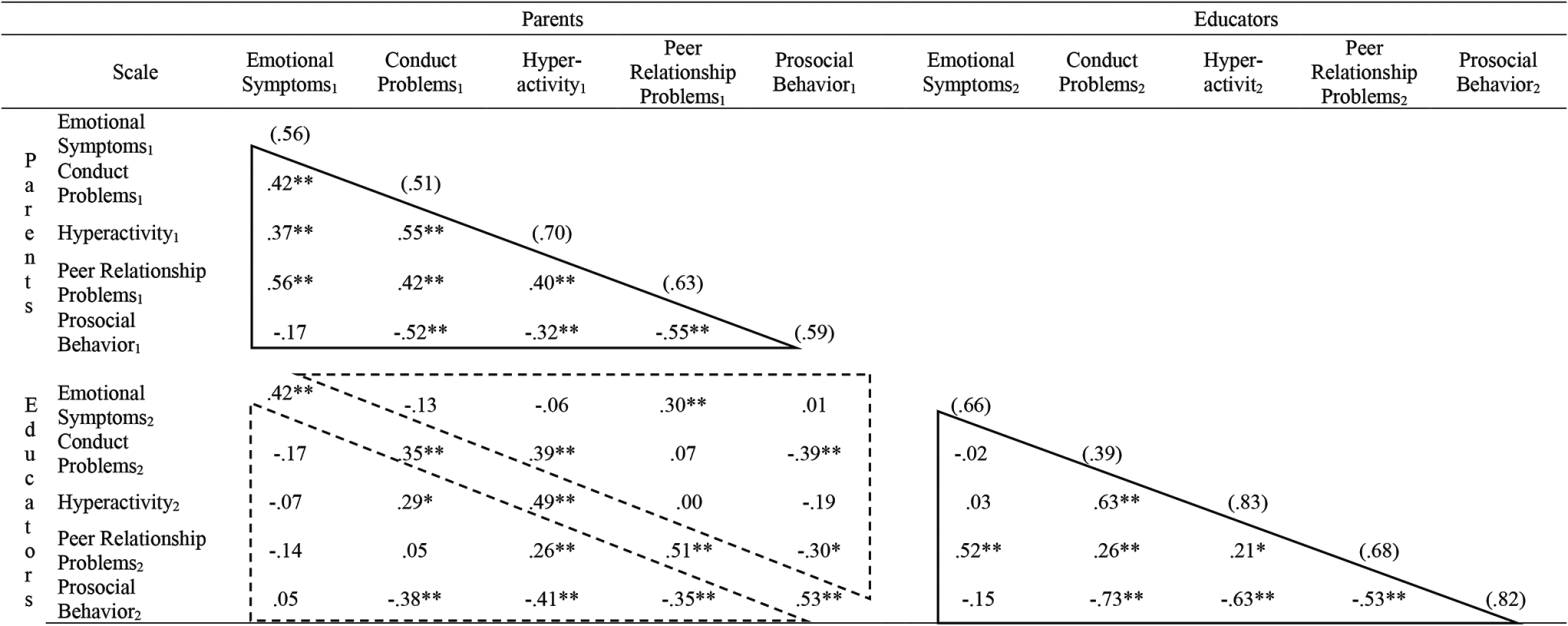
Results of the multitrait-multimethod matrix

*Note*: The values of the reliability diagonal in brackets refer to Cronbach’s alpha. All other coefficients denote polychoric correlations, conducted using structural equation models. The validity diagonal (dashed triangles) refers to monotrait-heteromethod correlations. Values in dashed triangles refer to heterotrait-heteromethod correlations, whereas values in solid-lined triangles refer to heterotrait-monomethod correlations. *p < .05; **p < .01; *n* = 241; Estimator = WLSMV; Model fit indices: RMSEA = 0.028 (90% CI = 0.02/0.03); CFI/TLI = 0.92/0.91; WRMR = 1.03. The subscales contain all given items of the questionnaire except for *pbullied* from the subscale peer relationship problems (parents) and *pworries* from the subscale emotional symptoms (parents) due to zero cells in their bivariate table

**Table 3:**
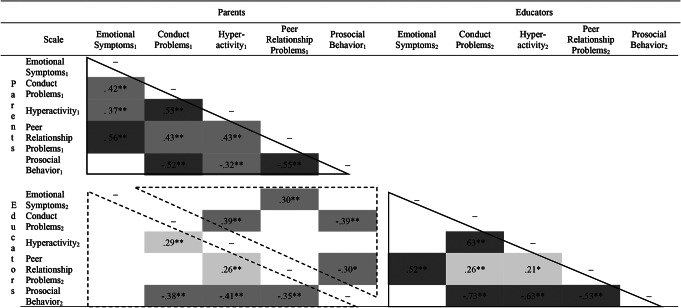
Visualization of significant coefficients of the multitrait-multimethod matrix

*Note*: Dark grey boxes visualize significant coefficients from *r* = .50-0.10; medium grey boxes visualize significant coefficients from *r* = .30-0.49; light grey boxes visualize significant coefficients from *r* = .10-0.29

The goodness-of-fit indices for the parents showed *RMSEA* = 0.05 (90% CI 0.04 − 0.06), *CFI* = 0.86 and *TLI* = 0.84 (*n* = 241), whereas the educators’ model showed model fit indices of *RMSEA* = 0.06 (90% CI 0.04 − 0.07), *CFI* = 0.94 and *TLI* = 0.93 (*n* = 157). Results show good indices for the model based on educators’ data structure, whereas parents miss the cut-off values marginally.

### Applying the Operations Triad Model (OTM)

In the theoretical section, we introduced the Operations Triad Model [39]. De Los Reyes [39] suggests that three criteria must be answered in the affirmative: Is a sufficient limit of reliability reached? Are findings sufficiently valid? Can methodological reasons be eliminated as explanations of divergence? If all criteria hold, we can interpret differences as meaningful findings instead of measurement errors [39]. Therefore, each subscale of the SDQ was examined according to its reliability and correlation coefficients (see Table 2):

Results show that the subscale Hyperactivity for educators (*α* = 0.83) and parents (*α* = 0.70) is sufficiently highly reliable and shows a moderate correlation between the two informants (*r* = .49) (see Table 2). Therefore, we attribute differences to meaningful, substantive differences in the children’s traits themselves, rather than measurement errors. Peer relationship problems show just acceptable high reliability, and the correlation between the two raters reaches a high correlation coefficient value. We attribute differences between raters to substantive, meaningful differences, rather than methodological causes. Emotional symptoms only show acceptable reliability for educators, whereas parents’ values are questionable. The correlation coefficient value ranges on a medium level. Due to missing quality criteria, findings on emotional symptoms might be due to methodological reasons and/or substantive trait differences. Conduct problems show the lowest values concerning internal consistency, and the correlation coefficient reaches a medium level. Before interpreting the findings, we would suggest investigating the assessed items more deeply.

We found significant mean differences between parents’ and educators’ prosocial behavior ratings. Cronbach’s alpha values for parents and educators differ, and the correlation coefficient shows the highest of all subscales. Also, prosocial behavior seems to have a broad general factor with conduct problems and hyperactivity. We conclude that both differences and agreements are due to substantive, not methodological reasons.

## Discussion

Screeners are a widely used tool to detect children’s emotional and behavioral strengths and difficulties. However, screeners should be collected from more than one informant (e.g., parents and educators) to provide a solid and holistic picture of the child. Parents and educators may assess children’s behavior differently, depending on the environment and their role toward the child. In addition, their perceptions are influenced by their relationship with the child and their characteristics. Therefore, parents’ and educators’ ratings may not necessarily coincide. In research, low rater agreement between different informants is a well-known phenomenon [2, 6, 10, 15]. In consequence, researchers assessing children based on parents´ and educators´ reports face the question of how to handle diverging findings. Our research question was based on these considerations.

We examined the agreements and differences between parents’ and educators’ perceptions of the five emotional and behavioral traits of the SDQ [45]. We applied t-tests and a multitrait-multimethod matrix and interpreted our results using the theoretical framework of the Operations Triad Model [39]. The model provides for considerations of reliability and validity of outcomes to distinguish between reasonable divergences and/or method effects [39].

Our results show that parents and professionals differ significantly on average in their perception of children’s prosocial behavior. Parents rated their children as having more prosocial behavior than the educators. The variation in the educators’ assessments is greater for the subscales of behavioral problems, hyperactivity, and prosocial behavior than for those of the parents. The findings show that educators assess the children in a more differentiated way than parents do. It could also be that children exhibit far more differentiated behaviors in daycare. Furthermore, it can be assumed that educators are more likely to have the opportunity to observe the child’s behavior in different activity settings and connections with varying peers than parents at home. Accordingly, the differences could also be explained by the different ways of interacting with the child and the associated different expectations towards the child.

Further findings of the MTMM-analysis and the interpretation using OTM show, we ascribe hyperactivity, peer relationship problems, and prosocial behavior to meaningful differences rather than measurement errors. Differences can be interpreted as varying child behavior. The assessment of hyperactivity is particularly reliable and shows a high level of agreement between parents and educators, which is why one perspective could be dispensed within studies. Therefore, we assume that hyperactivity is a relatively consistent behavior across different situations. Our results are in line with previous research [3, 51, 52]. The subscale prosocial behavior shows significant differences in mean values between parents and educators with high correlations at the same time. Because of the educator’s higher reliability and more homogenous reports on the children’s prosocial behavior, the subscale seems more appropriate for educators than for parents. We assume it is easier to observe prosocial behavior when interacting with peers than in the home setting. Additionally, educators shared professional experiences within the context and similar qualifications. The informant should be chosen thoughtfully when assessing prosocial behavior depending on the research question.

In contrast, we find differences in emotional symptoms and conduct problems due to measurement errors. With De Los Reyes et al. [39], compensating operations can therefore be assumed. Possible measurement errors should be taken into account when using these subscales.

Concerning hypothesis 2.2, it can be stated that there is no consistent discrimination of the traits across both perspectives, that of the parents and the educators, and thus the hypothesis is rejected. In particular, the subscales of the educators’ assessments of prosocial behavior, behavioral problems, and hyperactivity appear to be subject to a general factor. Goodman [16] himself advises combining subscales into an externalizing and internalizing factor.

Differences can have three reasons. First, different reference frames between educators and parents may be responsible for differences. Educators have daily contact with many children and are able to compare between children and have a clearer expectation of what is problematic or appropriate behavior. Secondly, children’s behaviors are perceived differently in different contexts. Externalizing behaviour is more likely to be perceived and age also plays a role. This could explain the high level of agreement between parents and educators’ reports on the hyperactivity subscale. Thirdly, significant differences can be attributed to the children’s role behavior and that of their educators. For example, educators are less able to respond individually to children in a group context, and children do not have the same expectations in this respect as they would have towards parents. For example, Fuertes et al. [53] showed, that parents and educators behave differently toward children in similar educational situations. While parents offer their children help and guidance, educators emphasize the child solving problems independently.

We assumed in advance that parents’ and educators’ ratings of the children differed and assessed our results based on divergent or compensatory operations. Another option is to assume converging ratings. For this option, an algorithm of the measure of agreement could be defined as proposed in the Operations Triad Model [39].

The results should be interpreted considering a few limitations. As the sample size is relatively small, interpretations should be made with caution. We used the survey’s entire data for parents to obtain a more reliable measure of the mean values and standard deviation. As a result, the sample sizes of parents and educators differ. As such, direct comparability is limited. Furthermore, the time interval between the parents’ and the educators’ ratings could have caused bias, as children’s development at a young age progresses quickly.

While previous research suggests variations in parental perceptions [52, 54], due to the small sample size, we were unable to conduct a meaningful analysis differentiating between maternal and paternal reports. This limitation underscores the need for caution in generalizing our findings to both parental perspectives.

Another important limitation of this work relates to constraints on generality. The educator and parent samples were predominantly European German women with global South and East European representation. Although our findings are consistent with the broader literature, our results are only generalizable to the German context and should be considered cautiously when extending to other societies. Conceivable characteristics for further investigation also in different social contexts could include the family’s socioeconomic status, poverty, ethnicity, race, or migration backgrounds.

While interpreting the results of this study, it is crucial to acknowledge the limitation associated with the educator‘s Cronbach alpha for the subscale conduct problems. The obtained alpha value indicates lower internal consistency for this aspect of our assessment. It could therefore be beneficial to exclude items from the analyses. However, these items differ depending on the perspective. Item structures were not changed in this study for analysis and interpretation to avoid making adjustments. It should also be noted that while Cronbach’s alpha remains a widely used and accessible measure of internal consistency, its limitations—particularly in the context of short scales and multidimensional constructs—necessitate cautious interpretation. This applies particularly to the parent’s scales of peer relationship and emotional symptoms.

Our study provides valuable insights into the assessment of children’s behavior using multiple informants, particularly parents and educators. The Operations Triad Model (OTM) provides a useful framework for systematically studying and comprehending informant discrepancies, which might reflect measurement confounds. The variability observed in informant reports underscores the importance of considering input from multiple sources when evaluating children’s social and emotional traits. This has significant implications for both research and practice in the field of child psychology and early childhood education.

In our analysis of the Strengths and Difficulties Questionnaire (SDQ), educators exhibited better model fit indices compared to parents. Despite aligning our structural equation models with Goodman et al.‘s (2010) framework, we faced challenges in achieving balance between parental and educator models, necessitating the exclusion of two indicators from the parental side model (items pbullied and pworries). To address this imbalance, researchers might consider revising certain SDQ subscale items or accounting for contextual factors that may influence assessments. For instance, items like pbullied and pworries may require special attention when diagnosing children with behavioral problems, as these may be more observable in an educational context than at home. While we acknowledge that excluding the two problematic items from both informant groups would enhance consistency across scales, we opted to retain them in the educator questionnaire to preserve valuable insights into the differential functionality of these items across informants. This decision, however, introduces a limitation regarding the comparability of the scales, which should be considered when interpreting the results.

Our findings highlight the need for a comprehensive approach to assessing children’s behavior, recognizing that different informants may offer unique perspectives based on their relationship with the child and the setting in which observations take place. This suggests that relying on a single informant may not capture the full range of a child’s behavior, emphasizing the importance of utilizing screening tools like the Strengths and Difficulties Questionnaire (SDQ) with input from both parents and educators. Furthermore, our identification of factors contributing to variability in informant reports, such as differences between home and preschool settings, suggests avenues for future research to explore these contextual influences in more depth. Understanding how these factors impact informant reports can inform the development of more effective interventions tailored to specific contexts and relationships.

For a more comprehensive understanding of the subject, we recommend further research on alternative instruments and diverse subsamples, including different language versions of the SDQ. Additionally, comparing various raters, such as informal caregivers or researchers themselves, to parents and educators could provide valuable insights. While predictors were not explored in our study, future research should investigate factors influencing parents’ and educators’ assessments of children’s behavior. Research questions could explore characteristics of children that predict similar or divergent ratings across multiple informants beyond those highlighted in our literature review, including externalizing versus internalizing behavior, gender, and age. Furthermore, future research directions could delve into personality traits like children’s self-efficacy, motivation, temperament, or resilience, examining interactions with child, parent, family, and educator characteristics. Particular attention could be given to exploring parent characteristics, especially within the parent-child relationship, despite the inherent challenges. Such investigations contribute to understanding the validity and reliability of parental statements, informing more effective intervention strategies.

Moreover, there is a need to investigate conditions predisposing infants to suboptimal development using multi-informant perspectives. For instance, Attention-deficit hyperactivity disorder (ADHD) is recognized as a heritable neurodevelopmental disorder, with symptoms manifesting consistently across settings. While genetic factors are central, early childhood trauma and developmental conditions may exacerbate symptoms or contribute to comorbid conditions [55–57]. Multi-informant perspectives from parents and educators are crucial for identifying genetic and environmental influences and facilitating more targeted early interventions and support strategies.

Practically, our findings underscore the importance of collaboration between parents and educators in supporting children’s social and emotional development. By recognizing and addressing discrepancies in informant reports, practitioners can gain a more comprehensive understanding of children’s behavior and tailor interventions accordingly. This collaborative approach aligns with the holistic nature of early intervention and education programs, which aim to support children’s development across multiple domains.

## Summary

Our study highlights the significance of considering ratings from multiple informants when assessing children’s behavior and emphasizes the need for a collaborative and comprehensive approach in research and practice. It is neither possible nor necessary to favor one group of informants over another. No gold standard can be set based on the assumption that both rater perspectives are justified depending on the research question. We suggest including at least one parent and educator in research studies using the SDQ. By recognizing and addressing variability in informant reports, children’s social and emotional well-being can be better supported and promote positive developmental outcomes.

## Data Availability

Data for this study are not publicly available. This study was not preregistered and has institutional review board approval.

## Footnotes

1 We refer to educatores as professionals working in early childhood education institutions throughout this article.
